# Effect of carbohydrate mouth rinsing on multiple sprint performance

**DOI:** 10.1186/1550-2783-10-41

**Published:** 2013-09-25

**Authors:** James L Dorling, Conrad P Earnest

**Affiliations:** 1 Department for Health, Sport, Health and Exercise Science, University of Bath, Eastwood 22-23 3.4, Bath BA2 7AY, UK

## Abstract

**Background:**

Research suggests that carbohydrate mouth rinsing (CMR) improves endurance performance; yet, little is known regarding the effect of CMR on multiple sprint efforts. As many sports involve multiple sprinting efforts, followed by periods of recovery, the aim of our current study was to investigate the influence of CMR on multiple sprint performance.

**Methods:**

We recruited eight active males (Age; 22 ± 1 y; 75.0 ± 8.8 kg; estimated VO2_max_ 52.0 ± 3.0 ml/kg/min) to participate in a randomly assigned, double-blind, counterbalanced study administering a CMR (6.4% Maltodextrin) or similarly flavoured placebo solution. Primary outcomes for our study included: (a) time for three repeated sprint ability tests (RSA) and (b) the Loughborough Intermittent Shuttle Test (LIST). Time was expressed in seconds (sec). Secondary outcomes included ratings of perceived exertion (RPE) and blood glucose concentration. Tertiary outcomes included two psychological assessments designed to determine perceived activation (i.e., arousal) and pleasure-displeasure after each section of the LIST. We analysed our data using a two-way analysis of variance (ANOVA) for repeated measures, a Bonferroni adjusted *post hoc* t-test to determine significant differences in treatment, and a liberal 90% confidence interval between treatment conditions. Effect sizes were calculated between trials and interpreted as ≤ 0.2 trivial, > 0.2 small, > 0.6 moderate, > 1.2 large, > 2 very large and > 4 extremely large. Data are means ± SD. Overall statistical significance was set as *P* < 0.05; yet, modified accordingly when Bonferroni adjustments were made.

**Results:**

Overall, we observed no significant difference in average (3.46 ± 0.2 vs. 3.44 ± 0.17; P = 0.11) or fastest time (3.38 ± 0.2 vs. 3.37 ± 0.2; P = 0.39) in the RSA test for the placebo vs. CMR conditions, respectively. Similar findings were also noted for the placebo vs. CMR, respectively, during the LIST test (3.52 ± 0.2 vs. 3.54 ± 0.2 sec; P = 0.63). Despite a significantly higher within group RPE during the 3rd and 4th sections of the LIST (< 0.05), no between group differences were otherwise noted. No differences were noted for blood glucose concentrations throughout the testing protocol. Lastly, from a psychological perspective, we observed no differences in pleasure-displeasure or perceived activation.

**Conclusions:**

The results of our current study suggest that CMR does not improve exercise performance, RPE or perceived pleasure-displeasure during high intensity activity requiring repeated, intermittent, sprint efforts.

## Background

It is well established that carbohydrate (CHO) ingestion improves prolonged (> 2 hours) steady-state [1] and intermittent endurance performance [2]. The proposed mechanisms for this ergogenic effect include a sparing of endogenous glycogen stores, an enhanced oxidation of exogenous CHO and the maintenance of high CHO oxidation rates during the later stages of exercise [3]. The ingestion of CHO before and during high intensity exercise over shorter durations (~ 1 hour) has also been found to enhance performance [4]. However, under these conditions, CHO ingestion exerts no influence on exogenous glucose uptake and total CHO oxidation [4]. To explain these findings, some authors hypothesize that CHO ingestion facilitates ergogenesis via the central nervous system, mediated by receptors in the oral cavity [5].

To investigate this theory, Carter et al. [5] examined the influence of mouth rinsing a CHO drink solution on time trial performance in nine cyclists. Interestingly, when compared to a placebo solution, mouth rinsing with a CHO solution resulted in a 2.9% improvement in performance [5]. Subsequent research has further demonstrated that carbohydrate mouth rinsing (CMR) enhances endurance performance during cycling [6] and running [7]. While others have reported contrary findings [8], research examining different exercise modes has indicated that CMR has no influence on maximal 30 sec sprint performance [9] or maximal strength [10].

Although the precise ergogenic mechanisms of CMR are not fully understood, Gant et al. [11] reported that mouth rinsing both sweet and non-sweet CHO enhanced motor evoked potentials to fresh and fatigued muscle by 9 and 30%, respectively. Other studies also suggest that CMR stimulates receptors in the mouth, which activate neural pathways to lower the perceptions of effort and improve subjective experiences during exercise [5]. Chambers et al. [6] provided support for this notion by demonstrating that CMR activates areas of the brain associated with reward and motivation using functional MRI.

Collectively, these findings raise the possibility that CMR may improve performance during multiple sprint exercise. To our knowledge, only one study has examined the influence of CMR on multiple sprint performance on a cycle ergometer [12]. Interestingly, Beaven and colleagues reported that CMR enhanced initial sprint performance, but also resulted in a greater performance decrement over their repeated sprint protocol [12]. Despite these findings, there is no published literature that has examined CMR during running activities which simulates multiple sprint sports. As such, further research would be useful to investigate whether CMR can provide an ergogenic benefit during a field test that replicates field-based team games. Furthermore, as previous research suggests an increased perception of exercise intensity may hinder performance during field-based team games [13], investigation of the influence of CMR on subjective experiences during multiple sprint exercise is required. The primary aim of our current study was to examine the effect of CMR on multiple sprint performance during a field-based exercise protocol. Secondary and tertiary aims included assessments regarding CMR on subjective experiences during multiple sprint exercise.

## Methods

### Participants

**Figure 1:**
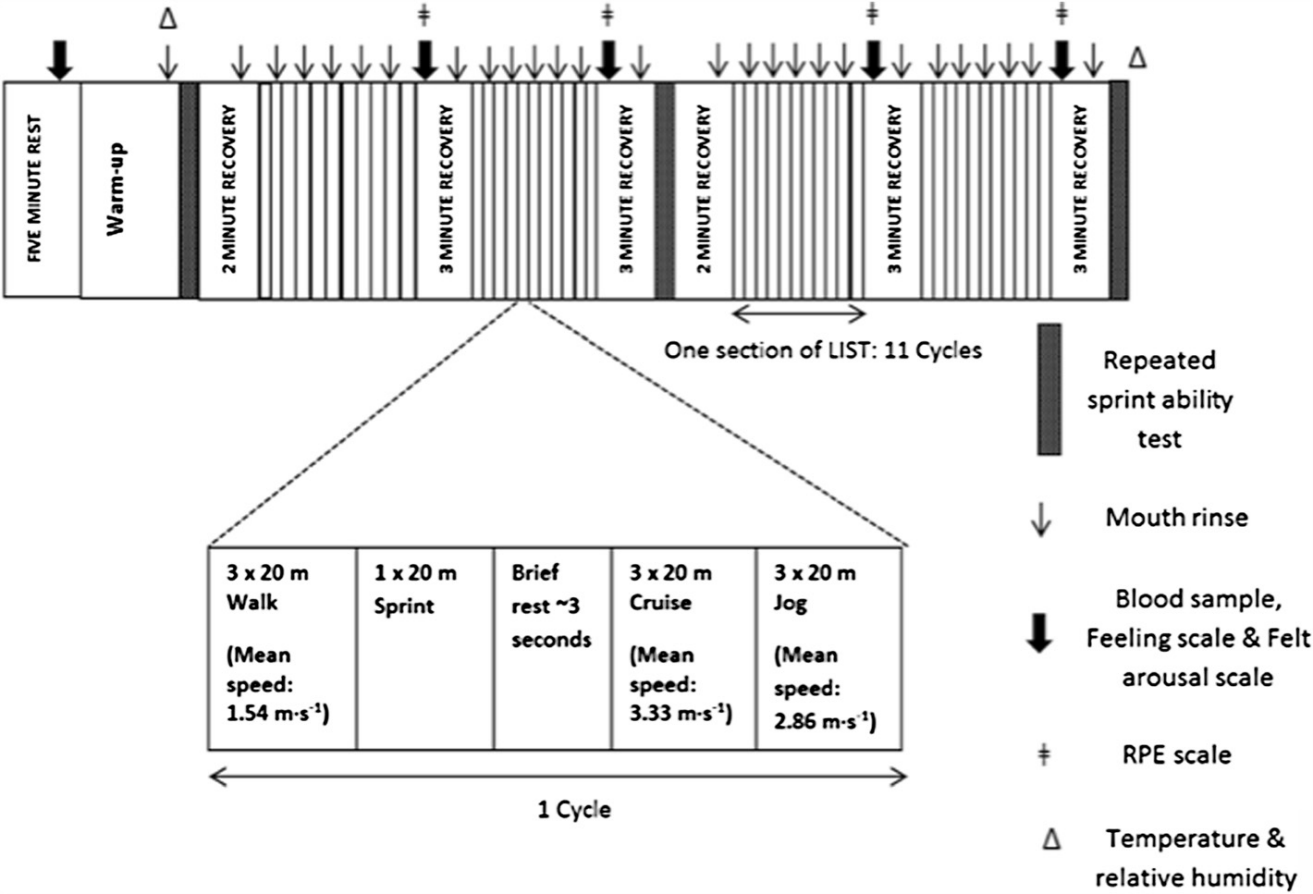
Schematic representation of the time line of study procedures.

Eight physically active males (Age; 22 ± 1 y; 75.0 ± 8.8 kg; estimated VO2_max_ 52.0 ± 3.0 ml/kg/min) volunteered to take part in the study. Seven of the participants habitually participated in field-based multiple sprint sport such as football (i.e., soccer) and rugby, while the other was a recreationally active runner. After participants were briefed about the nature of the study, they provided written informed consent. The exclusion criteria included usage of creatine supplements in the 12 weeks prior to the study, due to its influence on multiple sprint performance [14]. The ethics committee for the Department of Health at the University of Bath approved, which was according to the Declaration of Helsinki. We have presented a schematic representation of the experimental conditions is presented in Figure 1.

### Preliminary measures and test familiarization

Five days prior to the first experimental trial, participants reported to an indoor sprints track for preliminary measurements including the participant’s height and body mass. During this visit each participant completed a progressive multistage shuttle run test, which estimated maximal oxygen uptake [15]. Following this, each participant completed one 15 min section of the Loughborough Intermittent Shuttle Test (LIST) and one repeated sprint ability (RSA) test in order to familiarize themselves with the experimental tests. At the completion of this visit, participants were familiarized with the psychological scales used in this study.

### Experimental trials

During each experimental condition, participants completed two trials consisting of a CMR and placebo (PLA) supplement administered in a randomized, counterbalanced order. To maintain blinding to the investigators and participants, all treatments were pre-labelled and subsequently dispersed by a non-affiliated researcher not participating in this trial. Experimental trials were conducted 7-9 days apart and at the same time of day. In the 24 h preceding the first experimental trial, participants were asked to record their diet and then replicate it before the second trial. Participants were also asked to refrain from strenuous exercise and to abstain from caffeine and alcohol ingestion in the 24 h before each trial. On the day of the experimental trial, participants were asked to ingest 568 ml of water to maintain euhydration, and arrive in a fasted condition.

On the morning of each trial, participants presented at an indoor sprint track to perform a standardized warm up (10-min), which consisted of jogging, cruising, sprinting, dynamic stretching and the RSA protocol. This RSA was used as part of the warm-up and not as a measurement test. Temperature and relative humidity were recorded (Testo, Hampshire, UK) at the start and at the end of each experimental trial to check for changes in environmental conditions. Following the warm-up period, participants initiated the testing phase of the trial by performing the RSA test, followed by a 2-min recovery. Participants then completed the LIST [16].

The LIST was comprised of 15-min sections of intermittent shuttle running over a 20-m distance. Each section of the LIST consisted of 11 cycles of a set running protocol. One cycle was comprised of three 20-m walks (mean speed: 1.54 m · s^-1^), one 20 metre sprint, ~ 3 sec of rest, three 20 metre cruises (mean speed: 3.33 m · s^-1^) and three 20 metre jogs (mean speed: 2.86 m · s^-1^). Following each section, there was a 3-min recovery period. Appropriate speeds for the walk, cruise and jog shuttles of the LIST were dictated by audible signals from a pre-recorded disc. On completion of the 3-min recovery of the second and fourth section of the LIST, participants completed the RSA test, followed by 2-min recovery period (Figure 1). Throughout the experimental protocol, every attempt was made to ensure that the participants were not distracted. No interaction or encouragement occurred between the investigator and the participants, except for mouth rinse administration.

### Carbohydrate solutions

The CHO solution was a 6.4% maltodextrin solution, containing 64 g of maltodextrin (HighFive, Bardon, England) per 1000 ml of water. Maltodextrin was used because it is a non-sweet and colourless [5]. The PLA solution was water. To make solutions indistinguishable both treatments contained a non-calorific artificial sweetener consisting of sucralose (FlavDrops, MyProtein, Norwich, England). Each rinse solution was provided as a 25-ml bolus in a pre-weighed plastic cup. Participants were instructed to swirl all of the solution in their mouth for ~ 5 sec, before expectorating the solution back into the cup. Participants rinsed a solution 30 sec prior to each section of the LIST and each RSA test. Participants were also instructed to rinse a solution during the first 20 metre shuttle of the second, fourth, sixth, eighth and tenth cycles of each LIST section. In total, this equated to 27 rinses and 675 ml of solution being rinsed and expectorated during each trial (Figure 1). On completion of the study, participants were asked whether they could distinguish which solution contained CHO.

### Repeated sprint ability test and sprint measures

All 20 m sprints from the RSA test and the LIST were recorded using infrared timing gates (Smartspeed, Fusion Sport, Australia) and were commenced from a standing position 0.5 m from the first start gate. Individual sprint times of all 44 sprints of the LIST were recorded and the mean sprint time from each section was calculated.

The RSA test was comprised of four straight-line 20 m sprints, separated by 20 sec of active recovery. During the active recovery, participants were given verbal encouragement to jog back to the start line within ~ 10-12 sec. On return to the start line, participants were instructed to prepare for the next sprint. Following a three second countdown, participants were given the ‘go’ command, which instructed them to initiate the sprint. A hand-held stopwatch was used to monitor recovery time. From each RSA test, the fastest and mean 20 m sprint times were recorded. During the RSA test of the warm-up, sprint times were recorded and within-subject coefficient of variation was derived from six participants. The coefficient of variation for both the fastest time and mean time was 1.2%.

### Blood sampling and analysis

Blood glucose was measured to examine any potential metabolic effects of CMR. A capillary blood sample was taken at baseline and during each 3 min recovery period of the LIST. Blood samples were obtained in EDTA prepared tubes (Microvette 5000, Sarstedt, Leicestershire) and placed in ice. Following completion of the trial, blood samples were analysed in duplicate using an automated analyser (YSI 2300 Stat Plus, YSI, Yellow Springs, Ohio). The coefficient of variation for 10 replicates for blood glucose was 3.2%.

### Psychological scales

As a tertiary measure we performed a series of psychological inventory throughout the trial to assess the effects of CMR on the participant’s subjective experiences. The perceived activation scale (FAS) was used to assess the participant’s perceived arousal [17]. The FAS is a six-point measure ranging from 1 (*low arousal*) to 6 (*high arousal*). Backhouse et al. [18] reported the FAS as a valid measure when examining supplementation interventions. The feeling scale (FS) was used to measure the dimension of pleasure-displeasure [19]. The FS is an 11 point scale which ranges from -5 to +5. Anchors are placed at 0 (*neutral*) and at odd integers, ranging from +5 (*very good*) to -5 (*very bad*) [20]. The FS and FAS were administered at rest and immediately after each section of the LIST (Figure 1). The participant’s ratings of perceived exertion (RPE) were obtained using the Ratings of Perceived Exertion Scale [21]. The Ratings of Perceived Exertion Scale was administered immediately following each section of the LIST (Figure 1).

### Statistical analysis

Data were analysed using a two-way repeated measures ANOVA. If sphericity was violated, a Greenhouse-Geisser correction was applied for epsilon < 0.75, while the Huynh-Feldt correction was utilised for less severe asphericity (> 0.75). A Bonferroni adjusted *post hoc* test was used to locate variance, where significant statistical effects occurred.

Magnitude-based inferences were calculated for sprint measures to examine whether the differences between the CMR and PLA trials were meaningful [22]. Using a function of the *P*-value, *F*-value and degrees of freedom generated by an ANOVA, the effect of the intervention was expressed as 90% confidence intervals and likelihoods of whether the true effect indicated a positive, negative or trivial change in performance [22]. Cohen’s effect size [23] was calculated between trials for the three sprint measures: RSA test mean times, RSA test fastest times and the mean sprint times of the LIST. Effect sizes were interpreted as ≤ 0.2 trivial, > 0.2 small, > 0.6 moderate, > 1.2 large, > 2 very large and > 4 extremely large [24].

An effect was deemed unclear if the confidence intervals spanned both positive and negative thresholds for the smallest worthwhile effect, i.e., the effect could be beneficial or detrimental [22]. The smallest worthwhile change in sprint time was assumed to be 0.8% of the mean time for each sprint measure [25]. All results are means ± standard deviation (SD) or 90% confidence intervals when appropriate. Statistical significance was set as *P* < 0.05.

## Results

### Repeated sprint ability and Loughborough intermittent shuttle tests

**Figure 2:**
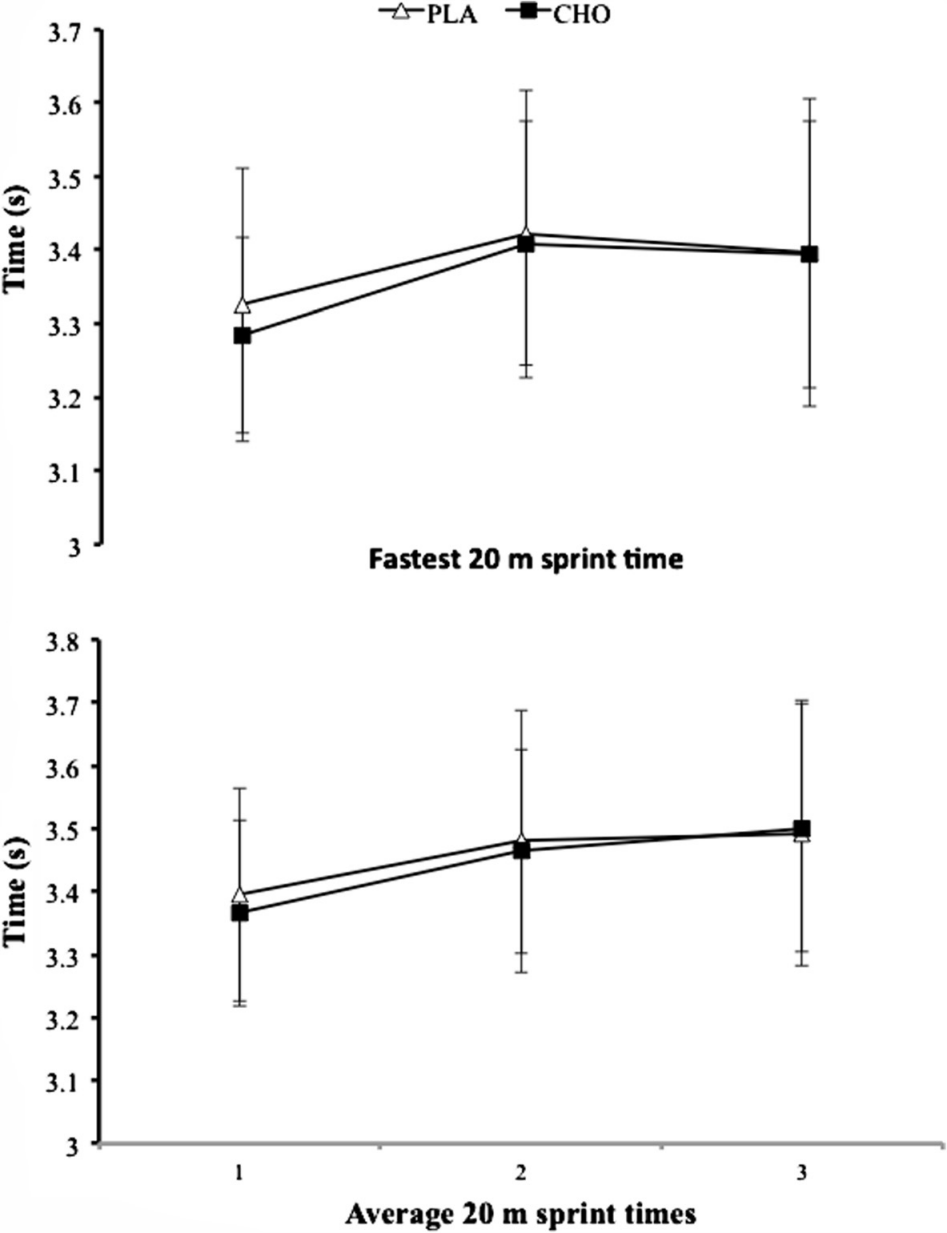
Data (mean ± SD) represent the fastest 20 m sprint time (top panel), and average 20 m sprint times (lower panel) for the RSA tests each experimental trial.

Throughout the testing protocol we observed no between trials for temperature (PLA, 21.9 ± 0.9°C; CHO, 22.0 ± 1.0°C; *P* = 0.84) or relative humidity (PLA, 60 ± 2%; CHO, 59 ± 3%; *P* = 0.43). With regard to the RSA, we observed a modest trend for the fastest sprint time of the RSA to increase throughout the trial as a whole; however, there was no main statistical effect for time (*P* = 0.07), treatment, or the time-by-treatment interaction effect (*P* = 0.56; Figure 2). The fastest sprint times of the RSA test were not significantly between treatment conditions for the CMR (3.37 ± 0.2) and PLA trial (3.38 ± 0.2 sec, *P* = 0.39). There were also no significant main effects of trial (PLA, 3.46 ± 0.19 sec; CHO, 3.44 ± 0.17 sec; *P =* 0.49), time (*P* = 0.11) and no interaction effect (*P* = 0.56) for mean RSA test time (Figure 2B). Although fastest sprint times of the RSA test tended to improve during the second trial (*P* = 0.09), there were no significant order effects for the three sprint measures (*P* > 0.05).

**Figure 3:**
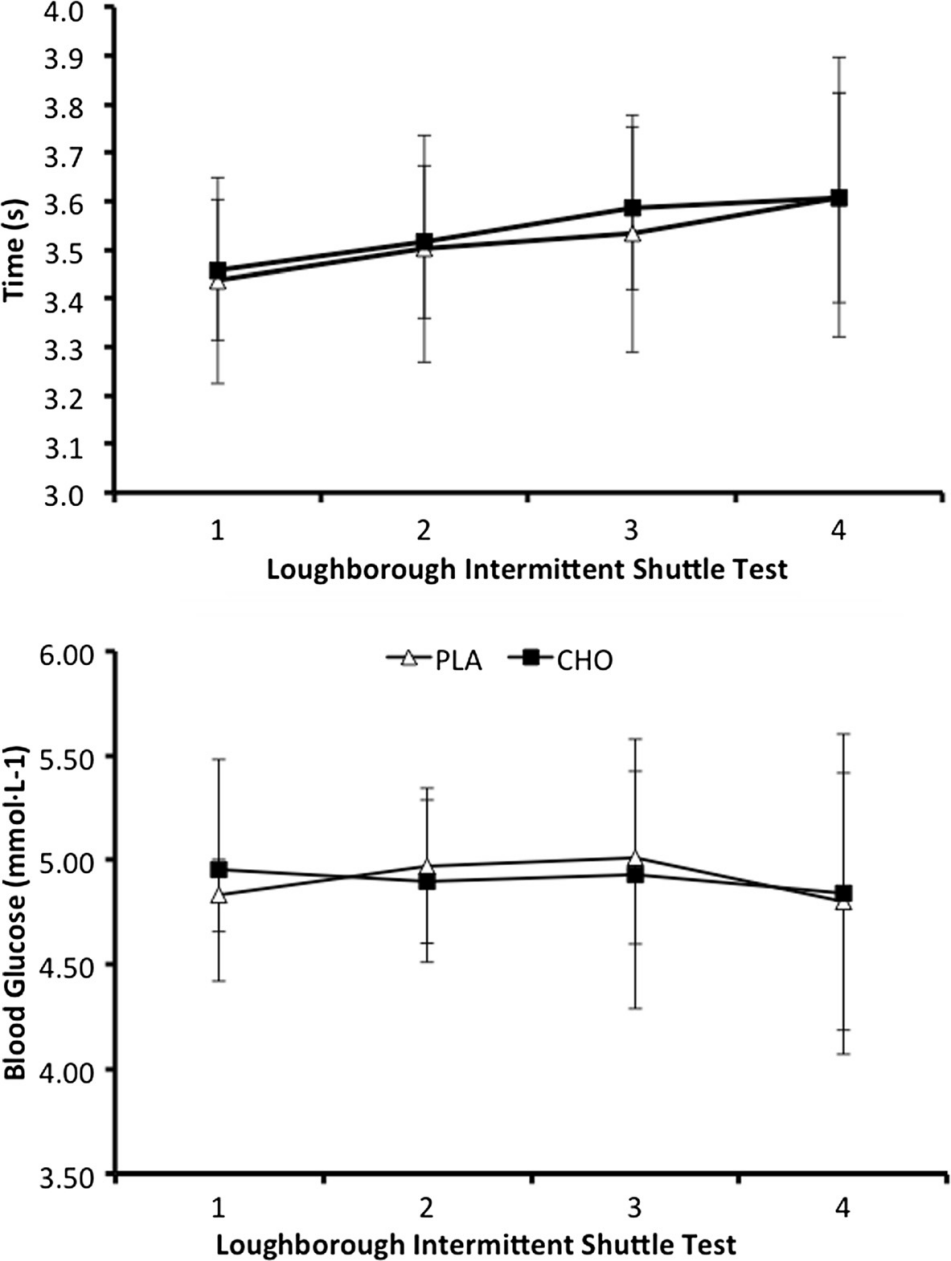
Data (mean ± SD) represent time (upper panel) and respective blood glucose concentrations (lower panel) observed during the LIST test.

Despite a significant effect of time (*P* = 0.001), showing an increase in sprint time throughout the LIST, there was no main effect of the treatment condition for the mean sprint times of the LIST (PLA, 3.52 ± 0.2 sec; CHO, 3.54 ± 0.2 sec; *P* = 0.63) and no interaction effect (*P* = 0.42; Figure 3). Finally, we observed no significant difference in blood glucose concentrations between trials (PLA, 4.90 ± 0.4 mmol · l^-1^; CHO, 4.90 ± 0.6 mmol · l^-1^; *P* = 0.78) and at no time point was blood glucose different (Figure 3). We deemed the effect sizes for all sprint measures as trivial ((≤ 0.2); Table 1). With regards to magnitude-based inferences, 90% confidence intervals overlapped the 0.8% smallest worthwhile effect for all sprint measures (Table 1).

**Table 1:** Absolute and standardized differences (effect size) between trials for sprint measures during the RSA and LIST tests

	**Absolute difference**	**Effect size**	**Percentage difference (90% confidence intervals)**	**Practical interpretation**
RSA average sprint time (s)	0.016 (↑)	0.09	0.5 (± 3.2)	Unclear
RSA fastest sprint time (s)	0.018 (↑)	0.10	0.8 (± 3.7)	Unclear
LIST average sprint time (s)	0.022 (↓)	0.10	0.3 (± 2.4)	Unclear

Percentage change with 90% confidence intervals and practical interpretations of magnitude-based inferences are also shown.

*Note:* Absolute differences are differences in mean. Upward (↑) and downward (↓) arrows represent whether the absolute difference is an improvement or decrement in performance when mouth rinsing CHO. Practical interpretations were considered unclear if 90% confidence intervals overlapped the smallest worthwhile change (0.8%).

### Psychological scales

We observed no significant effects of time on perceived pleasure-displeasure (FS; *P* = 0.033), but no differences were found between trials and no interaction effect was evident (*P* = 0.55; Table 2). We also observed no difference in perceived activation (FAS) between PLA and CHO trials (2.4 ± 1.2 vs. 2.5 ± 1.2, respectively; *P* = 0.28) and no effect of time (*P* = 0.25; Table 2). There was no main effect of trial on RPE (PLA, 13 ± 2; CHO, 14 ± 2; *P* = 0.84) or interaction effect. There was, however, a main effect of time on RPE (*P* = 0.001), with *post-hoc* tests revealing that RPE was greater following the third (*P* < 0.02) and fourth sections (*P* < 0.02) of the LIST, when compared to the first (Table 2).

**Table 2:** Scores for the FAS, FS and RPE during the CMR and PLA trials

				**Time point**		
**Scale**	**Trial**	**Baseline**	**Section 1**	**Section 2**	**Section 3**	**Section 4**
FS	CHO	1.1 ± 1.4	−0.3 ± 1.0	−0.8 ± 1.2	−1.1 ± 1.1	−0.9 ± 2.5
	PLA	1.4 ± 1.2	−0.1 ± 0.8	0.0 ± 0.5	−0.5 ± 0.9	0.0 ± 1.2
FAS	CHO	2.3 ± 0.5	2.6 ± 1.4	2.4 ± 1.3	2.5 ± 1.5	2.6 ± 1.2
	PLA	2.0 ± 0.8	2.6 ± 1.3	2.3 ± 1.2	2.4 ± 1.5	2.8 ± 1.4
RPE (6-20)	CHO	n/a	13 ± 1	13 ± 1	14 ± 2*	15 ± 2*
	PLA	n/a	12 ± 1	13 ± 1	14 ± 1*	14 ± 2*

* Significant within (i.e., time) effect noted for each group different to Section 1 (*P* < 0.05). No between group differences are otherwise noted. Data are mean ± SD.

## Discussion

The primary aim of the current study was to investigate the influence of CMR on multiple sprint performance. Primary measures included actual sprint times; while secondary measures examined RPE, blood glucose concentrations and psychological constructs of perceived activation and pleasure-displeasure. Our primary findings demonstrate that CMR does not improve intermittent high-intensity exercise performance as measured via the RSA and LIST. We also found that CMR had no effect on three subjective indices associated with exercise performance. Direct comparisons with the current literature are difficult as we are unaware of any published studies examining the influence of CMR during field-based multiple sprint performance. Nevertheless, the findings are broadly in line with those of Chong et al. [9] who reported trivial effect sizes of 0.01 - 0.14 for peak and mean power measures while examining the effect of CMR on sprint performance on a cycle ergometer. At odds with the current study’s findings, Beaven et al. [12] reported that CMR enhanced initial sprint performance during repeated cycle sprint exercise, but did not maintain power over multiple sprints. The precise reasons for this discrepancy are unknown but may be due to the increased demand of the protocol used in the current study. Indeed, as the current protocol, including the warm up, was used to simulate field-based team game activity, the increased number of sprints may have led to other overruling factors that caused fatigue to accrue. Specifically, other mechanisms of fatigue seen during team-game sport such as alterations in intramuscular phosphates and the reduction in phosphocreatine may have negated any ergogenic influence of the CMR [26,27]. Though this notion requires further research, it is supported by Jeukendrup and Chambers [28] who suggested that the mechanisms, which cause fatigue during intense activity, may nullify any performance enhancing effects of CMR.

Many studies which report an ergogenic benefit while using CMR postulate that the presence of CHO in the oral cavity triggers receptor cells in the mouth, which stimulate reward centres in the brain such as the orbitofrontal cortex and the ventral striatum [6]. In turn, this stimulus may lower perceptions of effort and/or improve motor output without an increase in perceived exertion [5]. In the current study, mouth rinsing CHO elicited no reductions in RPE or any evident dissociations between motor output (sprint times) and RPE. This is at odds with studies that report CMR augments exercise intensity for a given RPE score [5] and decreases RPE for a given absolute work rate [29]. Although further research is warranted to fully elucidate this difference, the results from the current study may suggest that CMR is incapable of reducing perceived exercise intensity during multiple sprint exercise. Of course, as the oral sensing of CHO may be just one of a large number of physiological and psychological inputs which modify RPE during multiple sprint activity [30], any reduction in perceived exertion due to CMR is perhaps negligible.

Further to the effects on perceived intensity, it has been proposed that CMR may improve the subjective evaluation of ‘how one feels’ during exercise [7]. The current study administered the FAS and FS to assess feelings of perceived activation and pleasure-displeasure, respectively. Results from the current study suggest that CMR was unable to improve perceptions of pleasure and activation. In contrast, Rollo et al. [7] reported that CMR increased feelings of pleasure during the first five minutes of a 30 min running procedure. Discrepancies between these findings are likely to be due to the different demands of the exercise protocols. Specifically, the aim of Rollo and colleagues protocol was to sustain a pace, which denoted a rating of 15 on the RPE scale [7], while the current study required participants to perform the sprints of the LIST and RSA tests. Perhaps, as optimal performance in the current study required participants to perform maximally during the sprints, the overriding motivation to perform well may have negated any small changes in the feelings of pleasure-displeasure and activation induced by the presence of CHO in the oral cavity [30]. In addition, any central changes caused by CMR may be evident for multiple sprint activity of 60 min or greater in duration. Though further research is required to confirm this notion, it may be supported by Backhouse et al. [18] who reported that CHO ingestion only improves perceived activation between 60 and 90 min of the LIST protocol.

Hypothetically, Carter et al. [5] suggest that CMR results in a cephalic rise in insulin and blood glucose, which improves performance by facilitating glucose uptake into the muscle. Contrary to this postulation, our current study indicates that CMR exerts no effect on blood glucose during multiple sprint exercise. This agrees with previous literature reporting that CMR has no influence on blood glucose concentrations during endurance exercise [31]. Although we did not measure peripheral changes in metabolism in our current study, our results support to the notion that CMR exerts little or no metabolic changes.

Despite the relatively small sample size of our study, we are confident in our findings. A major strength of our current study is that it represents a fairly “real world” testing scenario synonymous with sport as the LIST correlates well with soccer and hockey performance [16,32]. Overall, we used a randomized, crossover treatment assignment to CMR and placebo conditions, whereby participants in our study served as their own controls.

The results of our RSA test coefficient of variations for fastest and mean sprint time (1.2%) were similar to other studies using RSA tests [33] and LIST [16]. The trivial effect sizes between trials questions whether there is any ergogenic influence of CMR on multiple sprint performance. We also observed very low coefficients of variation between testing each testing condition (*all*, < 2.0%). Thus, our study was additionally robust owing to the small variance that we observed between testing conditions, which ultimately attest to the reliability of our study protocol. Finally, though it is more common to use 95% confidence intervals, our use of fairly broad confidence intervals (90%) to detect magnitude of effect based inferences revealed that the influence of CMR on sprint times of the RSA test and LIST were unclear as suggested Batterham and Hopkins [22]. An additional strength of our study was ability to control temperature and relative humidity during testing conditions as environmental factors have been found to influence sprint performance [34].

In spite of these strengths, the current study has limitations. First, there was no procedure used to ascertain whether any CHO or fluid was ingested, such as measuring the expectorant to equate mouth rinse “ingestion” with expulsion. Though the blood glucose concentrations were similar between trials, there was insufficient time in the testing facility to reweigh each expectorated solution to establish absolutely whether any CHO or fluid was inadvertently ingested. Second, due to size and homogeneity of the sample studied, we are unable to generalize our results to other populations. Third, one criticism of our study is that we tested participants in a fasted state, which is at odds with training and competition. However, Lane et al (2013) have shown that CMR in the fasted state improves performance more so than a fed state [35]. Therefore, our results are not likely confounded by a fed vs. fasted treatment condition.

Finally, though the LIST is designed to be a field test emulating soccer performance, it does not adequately account for various time points during a match. Therefore, it may be worthwhile to assess CMR under more match appropriate time conditions such as at the beginning, half way point (~ 45 min) and ~90 min) of exercise.

## Conclusions

On the whole, results from our current study suggest that CMR exerts no influence on multiple sprint performance during a field-based test designed to simulate team game sports. Though our results suggest that CMR is an ineffective ergogenic aid during field-based activity, further confirmatory study is required to examine CMR during time periods more applicable to team game sports and to investigate CMR following a period of preload.

## Competing interests

The authors declare that they have no competing interests.
